# Two-dimensional Graphene/MoS_2_ vertical heterostructure for detection of hemoglobin concentration in blood samples

**DOI:** 10.1371/journal.pone.0310166

**Published:** 2024-09-10

**Authors:** Manoj Kumar, Purnendu Shekhar Pandey, Vivek Kumar Srivastava, M. Sudhakara Reddy, Anita Gehlot, Yadvendra Singh, Gyanendra Kumar Singh, Balkeshwar Singh

**Affiliations:** 1 MLR Institute of Technology, Hyderabad, India; 2 Department of Electronics and Communication Engineering, GL Bajaj Institute of Technology and Management, Greater Noida, U.P., India; 3 Electrical Engineering Department, GLA University, Mathura, India; 4 Department of Physics & Electronics, JAIN (Deemed to be University), Bangalore, Karnataka, India; 5 Uttaranchal Institute of Technology, Uttaranchal University, Dehradun, India; 6 Laxmi Chand Institute of Technology, Bilaspur, India; 7 Department of Mechanical Engineering, Program of Manufacturing Engineering, Adama Science and Technology University, Adama, Ethiopia; International Iberian Nanotechnology Laboratory, PORTUGAL

## Abstract

This study demonstrates the use of computational methods to simulate the molecular dynamics involved in hemoglobin concentration sensing, utilizing Material Studio and the TCAD Silvaco device simulator. A non-invasive and flexible Graphene/MoS_2_ heterostructure has been proposed for sensing hemoglobin concentration in blood samples. The findings reveal a notable shift in the wavelength-dependent refractive index and extinction coefficient, as well as significant changes in the absorption coefficient and reflectivity of the Graphene/MoS_2_ heterostructure in response to different hemoglobin concentrations, specifically within an approximate range of 0.3 μm to 1 μm. Moreover, the spectral response of the heterostructure demonstrates that at a particular wavelength of approximately 600 nm, a maximum response is obtained. This wavelength can be considered optimal for detecting various levels of hemoglobin using this heterostructure. The anticipated outcome is a comprehensive understanding of the fundamental principles, ultimately resulting in the development of an exceptionally sensitive platform for detecting hemoglobin concentration.

## 1. Introduction

In the field of medical diagnostics, early disease detection and effective healthcare management greatly depend on the timely and accurate identification of biomarkers. Hemoglobin is an important marker for a variety of hematological diseases and disorders. It is an essential protein that is vital to the circulation’s ability to carry oxygen. Anaemia, sickle cell disease, and hemoglobinopathies are just a few of the illnesses for which accurate and sensitive hemoglobin level detection is crucial to the diagnosis and treatment process. Recently, the use of nanotechnology and two-dimensional materials (2D) to biological research has opened up new avenues for the creation of detection systems with extraordinary sensitivity and efficiency [1, 2]. Graphene and molybdenum disulfide (MoS_2_) have attracted considerable interest within the realm of 2D materials owing to their distinctive characteristics and ability to interact with flexible substrates [3–5]. The present study investigates the possibility of a new flexible heterostructure composed of 2D materials such as Graphene and MoS_2_ as an advanced platform for the detection of hemoglobin concentration in blood samples. The synergistic heterostructure formed by the combination of Graphene, which has exceptional electrical, mechanical, and thermal characteristics, with MoS_2_, a semiconducting material, presents an opportunity for better sensing capabilities [6–8]. The inherent flexibility 2D materials, Graphene/MoS_2_ heterostructure may facilitates the development of wearable and point-of-care devices, offering a non-invasive and simple method for monitoring hemoglobin levels [9, 10]. Through harnessing the distinctive optical characteristics of Graphene/MoS_2_ heterostructure, we expect to develop a detection technique that is both very sensitive and selective, surpassing current constraints [1, 11, 12], which include compatibility of hemoglobin level detection sensors with existing healthcare systems and electronic health records for seamless data integration and patient care. When incorporated into wearable devices, these sensors need to be compact, durable, and capable of reliable long-term operation. However, developing such accurate, reliable, and non-invasive sensors can be costly, potentially limiting their accessibility, particularly in low-resource settings. Additionally, while non-invasiveness is a key goal, some sensor designs still require physical contact with the skin or other body parts, which can be uncomfortable or inconvenient for continuous monitoring. In order to overcome aforementioned limitation, we aim to propose a flexible and highly responsive, contactless hemoglobin level detection [13, 14]. This finding has the potential to have a significant effect that goes beyond only detecting hemoglobin concentration. It can lead to the creation of sophisticated diagnostic tools that can be used in customized medicine and point-of-care diagnostics [15]. Moreover, nano-confinement effect can provide great opportunities and improved functions in 2D materials which enables light confinement at the nanoscale, often represents interactions between light and 2D materials, which basically alters its wavelength dependent optical properties such as refractive, extinction and absorption coefficient [16, 17]. Although, there has been a lot of study in the area of sensors on two-dimensional materials, which may be utilized to change sensor surfaces to boost sensor sensitivity [18, 19]. The application of Material Studio and TCAD in this work advances the understanding of molecular interactions and facilitates the prediction and analysis to enhance implications of the findings which extend beyond the identification of hemoglobin concentration. They offer a framework through which computational modelling can be integrated into the creation and improvement of biosensing platforms that are employed in numerous biomedical applications.

## 2. Design consideration

**Fig 1:**
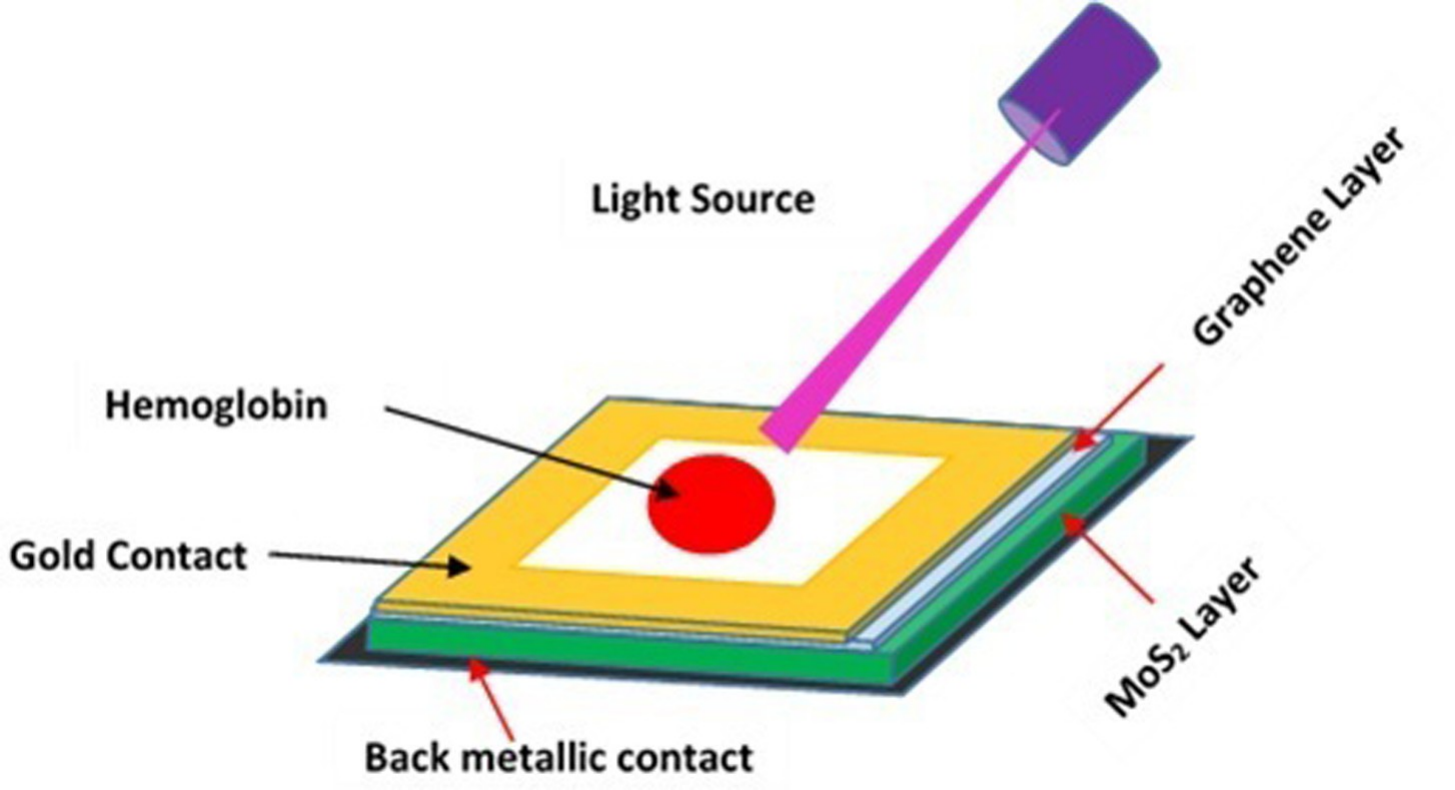
Schematic design of Graphene/MoS_2_ heterostructure for hemoglobin detection.

The schematic of Graphene/MoS_2_ heterostructure for detection of hemoglobin concentration has been shown in Fig 1. The hemoglobin molecule present in blood sample to be detected is placed on the top of Graphene/MoS_2_ heterostructure. This structure is backed by two electrode front (gold contact) and back (metallic contact) electrode (shown in Fig 1).

**Fig 2:**
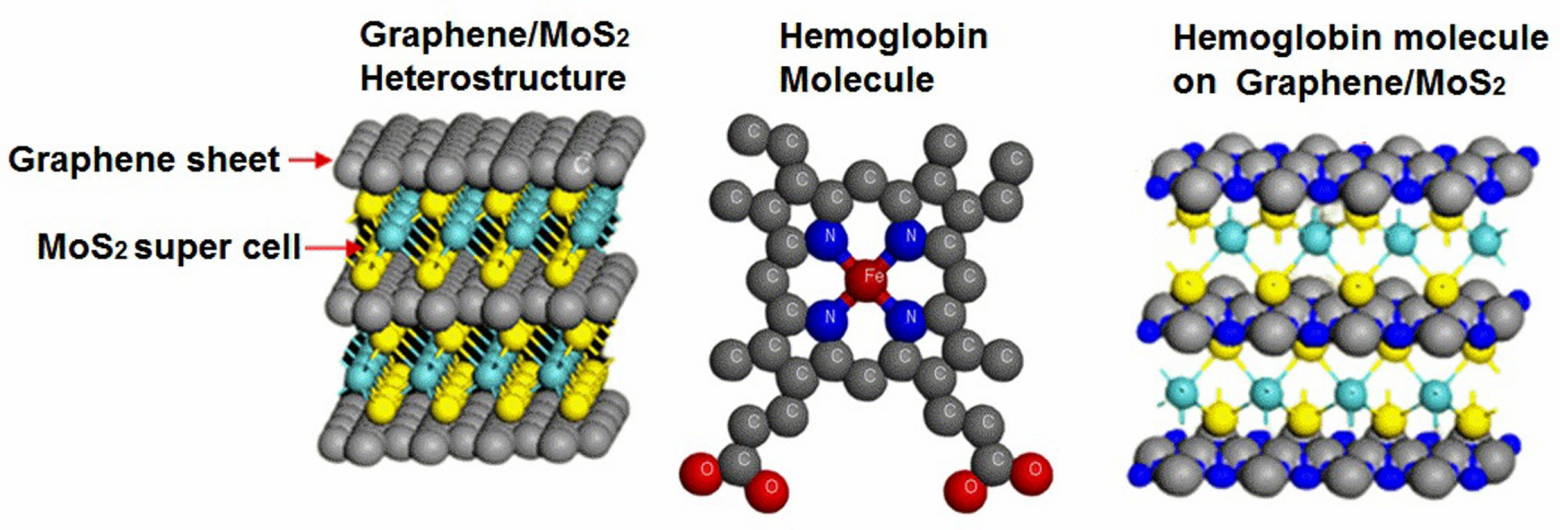
Molecular representation of Graphene/MoS_2_ heterostructure, hemoglobin molecule and absorption of hemoglobin molecule on Graphene/MoS_2_ heterostructure.

Proceeding to computational modeling of aforementioned heterostructure, a MoS_2_ super cell (3×3) was built in conjunction with Graphene nanosheet which leads to the formation Graphene/MoS_2_ heterostructure as shown in Fig 2. The Fig 2, from left to right shows molecular representation of Graphene/MoS_2_ heterostructure, hemoglobin molecule and absorption of hemoglobin molecule on surface of Graphene/MoS_2_ heterostructure [20].

It may be mentioned here that in this work, different hemoglobin molecular concentration has been referenced as hemoglobin molecule-I, hemoglobin molecule-II and hemoglobin molecule-III. The hemoglobin molecule-I indicates relatively lowest concentration and hemoglobin molecule-III represents highest concentration, whereas hemoglobin molecule-II denotes a mid-range concentration of hemoglobin. In this work, the CASTEP toolkit package of Material Studio were employed in order to obtain different tunable optical properties of Graphene/MoS_2_ super cell with change in concentration of hemoglobin molecules. The calculations were performed using generalized gradient approximation (GGA) with the Perdew-Burke-Ernzerhof (PBE) functional by norm-conserving pseudopotentials of Cambridge Sequential Total Energy Package code (CASTEP) tool kit of Material Studio [21]. The convergence tolerance parameters value were set to be as 830 eV as cutoff energy for the k-point fine mesh of 9×9×2, maximum force, maximum stress and maximum displacement were set to be 0.03 eV/A^0^, 0.05 GPa and 0.001 A^0^, respectively. The SCF tolerance value was considered to be fine, i.e., 10^−9^ eV/atom. The amount of hemoglobin present on the surface of Graphene/MoS_2_ heterostructure was varied and correspondingly change in optical properties of heterostructure such as refractive index, extinction and absorption coefficient were observed. Thereafter, obtained wavelength dependent optical properties of Graphene/MoS_2_ heterostructure with change in content of hemoglobin were used in Technology Computer-Aided Design (TCAD, Silvaco) device simulator to obtain the spectral responses. These spectral responses of aforementioned heterostructure helps to understand its response or sensitivity for particular wavelength of incident light [22, 23].

## 3. Results and discussion

**Fig 3:**
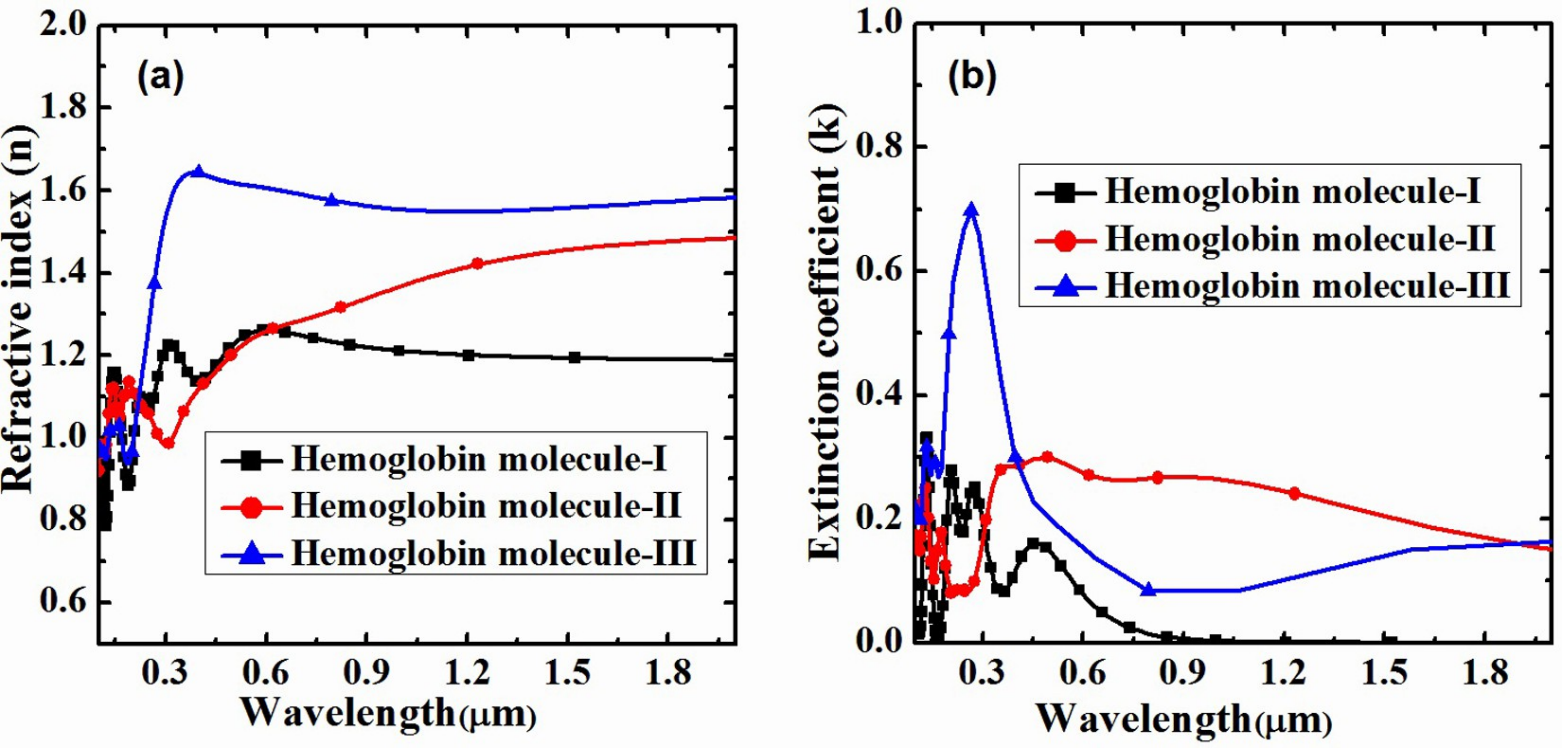
Variation of refractive index and extinction coefficient with change in concentration of hemoglobin on the surface of Graphene/MoS_2_.

The investigation into the `detection of hemoglobin concentration utilizes a flexible heterostructure composed of Graphene/MoS_2_, in conjunction with the computational modeling capabilities of Material Studio and TCAD, yielded noteworthy findings regarding the fluctuations in refractive index, extinction, and absorption coefficient in relation to alterations in the concentration of hemoglobin molecules. The obtained results indicate a significant change in the refractive index of the Graphene/MoS_2_ heterostructure with increasing hemoglobin concentration. The relationship between the wavelength (in micrometers) and the refractive index (n) for three different hemoglobin molecules—hemoglobin -I, hemoglobin -II, and hemoglobin -III has been shown in Fig 3(A). The observed fluctuations in refractive index exhibit a robust correlation with the gradient of hemoglobin content, suggesting a feasible approach for the quantitative detection of hemoglobin level [24, 25]. The refractive index of hemoglobin molecule-I is comparatively constant, averaging 1.1 over the wavelength range with just slight variations. Beginning just above 1.0 at 0.3 μm, hemoglobin molecule-II exhibits a continuous rise that peaks at 1.0 μm at about 1.35 before stabilizing. Significant fluctuation is seen in hemoglobin molecule-III, which begins at 1.4 at 0.3 μm, rises quickly to 1.65 at 0.4 μm, then progressively decreases and stabilizes at 1.4 at 0.9 μm and beyond. These variances imply different optical behaviors, most likely resulting from changes in molecule composition and structural configuration because of different amount hemoglobin molecules on the surface of Graphene/MoS_2_ heterostructure.

**Fig 4:**
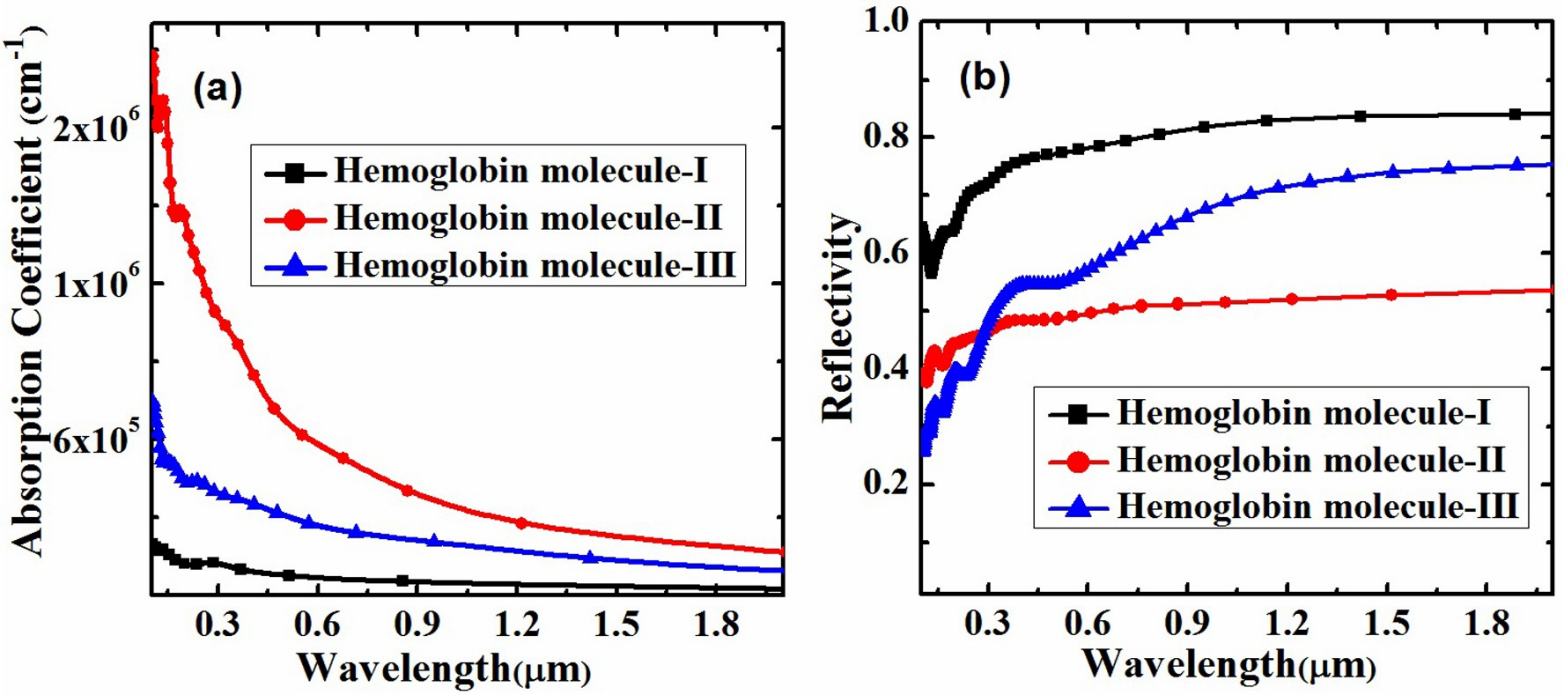
Variation of absorption coefficient and reflectivity with change in concentration of hemoglobin on the surface of Graphene/MoS_2_ heterostructure.

The computational investigation reveals that the extinction coefficient and absorption coefficient (shown in Figs 3(B)) and 4(A), respectively) of the Graphene/MoS_2_ heterostructure undergo noticeable variations when exposed to different hemoglobin concentrations. The observed variation in extinction and absorption coefficients provide a measurable and distinguishable response to changes in hemoglobin concentration, hence enhancing the sensitivity of the detection platform. The extinction coefficient (k) for each of the three types of hemoglobin molecules against wavelength (measured in micrometres) is shown in Fig 3(B). The wavelength is shown by the x-axis, which spans around 0.2 μm to 1.8 μm, and the extinction coefficient is displayed by the y-axis, which ranges from 0 to 1. At shorter wavelengths, hemoglobin molecule-I shows notable extinction coefficient values, peaking at 0.3 μm and rapidly declining with increasing wavelength. On the other hand, hemoglobin molecule-II has a more widely distributed peak, centered at 0.4 μm, and sustains a comparatively elevated extinction coefficient between 0.4 μm and 0.8 μm before progressively declining. The third and highest peak, again at approximately 0.3 μm, is exhibited by hemoglobin molecule-III. Its extinction coefficient declines more gradually and stays substantially higher across a wider wavelength range, reaching approximately 0.9 μm. For all three types of hemoglobin molecules, the wavelength dependence reveals that the extinction coefficient is strongest at shorter wavelengths and diminishes with increasing wavelength. The different light absorption patterns among the compounds suggest structural or compositional variations that impact how the molecules interact with light which clarifies the various ways that hemoglobin molecules absorb light. Moreover, the absorption coefficient for each of the three types of hemoglobin molecules as a function of wavelength (measured in micrometers) is illustrated in Fig 4(A). It is observed that the absorption coefficient, which is highly correlated with the extinction coefficient, hemoglobin molecule-II exhibits comparatively higher absorption value as compared to hemoglobin molecule-I and hemoglobin molecule-III. This is due to the fact that hemoglobin molecule-II exhibiting the greatest extinction and absorption coefficients which is attributed to its lower surface reflectivity [26], compared to hemoglobin molecule-I and hemoglobin molecule-III, as shown in Fig 4(B). Furthermore, the variation in surface reflectivity (shown in Fig 4(B)) of Graphene/MoS_2_ may be attributed to stoichiometry and molecular orientation of the hemoglobin molecules on its surface [27].

**Fig 5:**
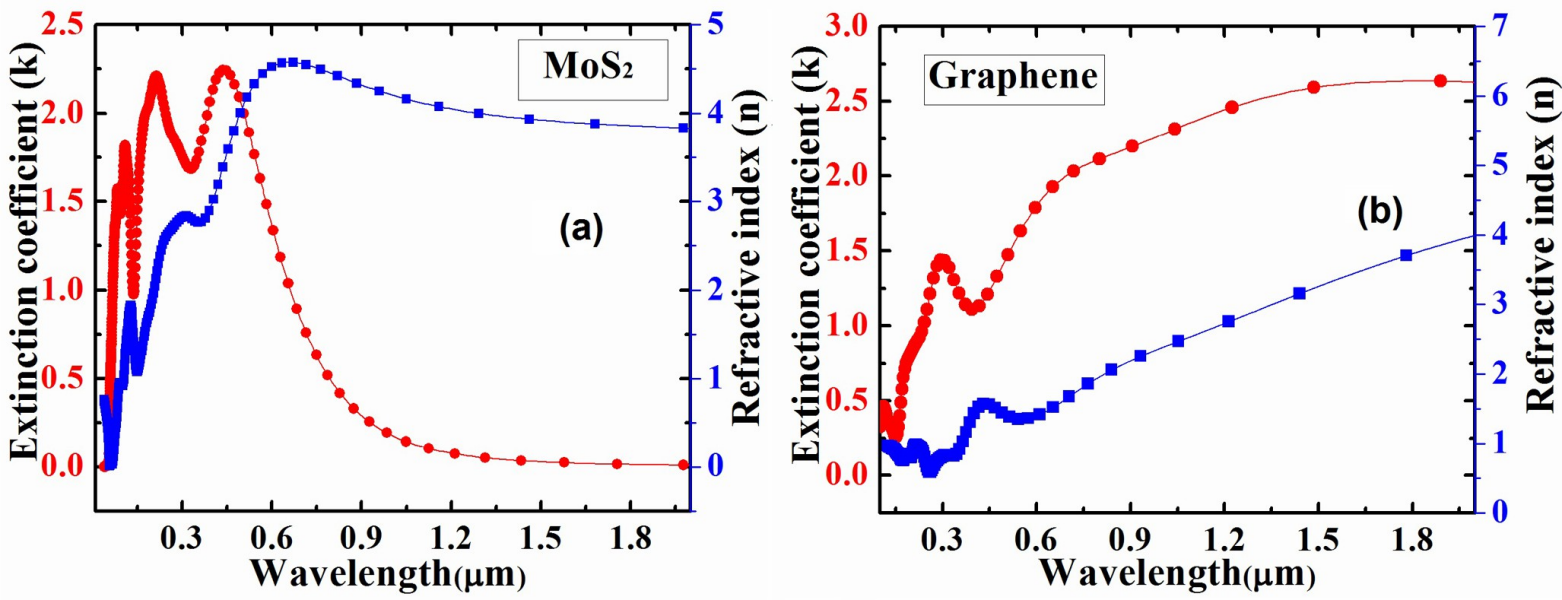
Wavelength dependent extinction coefficient and refractive index of MoS_2_ and Graphene.

Finally, TCAD (Silvaco) device simulator was used to calculate the spectral response in order to find response of Graphene/MoS_2_ heterostructure in conjunction with different concentration of hemoglobin molecule for different wavelength of incident light. The material parameters such as wavelength dependent refractive index and extinction coefficient of MoS_2_ and Graphene as shown in Fig 5(A) and 5(B), respectively, have been used for TCAD simulation to obtain the spectral response of Graphene/MoS_2_.

**Fig 6:**
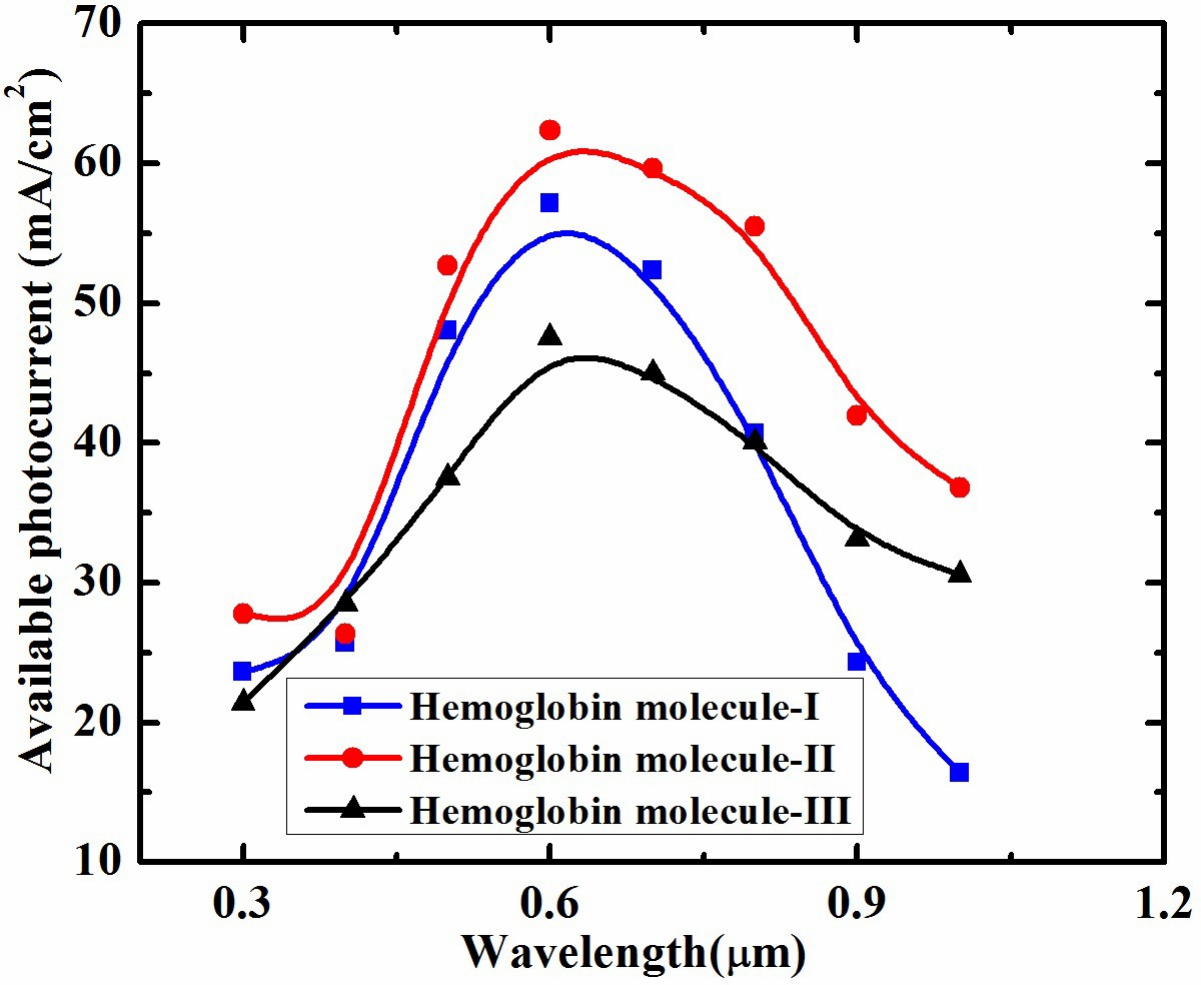
Spectral response of Graphene/MoS_2_ heterostructure with change in concentration of hemoglobin.

The spectral response (shown in Fig 6) of heterostructure reveals that for a particular wavelength, i.e., ~600 nm, maximum response is obtained which can be considered as optimized wavelength for this heterostructure for different level of hemoglobin detection. Using a light source with a wavelength of approximately 600 nm, it is possible to detect hemoglobin concentration. Fig 6 clearly shows that different hemoglobin concentrations in blood can be measured using this optimized wavelength.

It may be mentioned here that spectral response at different wavelength of light has been calculated in terms of available photocurrent is represented by Eq (1). The available photocurrent can be thought of as a measure of the photo absorption rate, expressed in terms of current density which is expressed as [28, 29].


$$I_{A}=\frac{qBn\lambda }{hc}\sum_{i=1}^{N_{R}}W_{R}\int_{0}^{Yi}P_{i}\alpha_{i}e^{\left(-\alpha_{i}y\right)}dy$$ (1)

Where I_A_ is available photocurrent, N_R_ stands for number of rays traced and W_R_ is width associated with incident rays.

The integral is taken over the length, *Yi*, associated with the ray, whereas *Pi* accounts for the attenuation and *α*_*i*_ is absorption coefficient.

## 4. Conclusion

This research demonstrates how computational methods can replicate the molecular dynamics of hemoglobin concentration in blood samples using software like Material Studio and TCAD in order to understand the complex relationship between various hemoglobin concentrations and the optical properties of the Graphene/MoS_2_ heterostructure through advanced computational techniques. Moreover, this study specifically examines the impact of changes in hemoglobin concentration on the refractive index, extinction coefficient, absorption coefficient and spectral response using Graphene/MoS_2_ heterostructure. It has been observed that at shorter wavelengths, changes in hemoglobin concentration result in a notable increase in the absorption and extinction coefficients, peaking at around 0.3 μm and rapidly declining as the wavelength increases. However, the refractive index and surface reflectivity of the heterostructure show significant changes at wavelengths greater than 0.3 μm with an increase in hemoglobin concentration. The spectral response of the heterostructure, illustrates that the maximum response occurs at a specific wavelength of approximately 600 nm. This wavelength can be considered optimal for detecting different levels of hemoglobin. Finally, studying these optical property changes, the Graphene/MoS_2_ heterostructure allows us to gain a comprehensive understanding of the fundamental concepts, paving the way for developing a highly sensitive and accurate hemoglobin concentration detection platform.

## Supporting information

S1 Data (RAR)
